# Targeting DCLK1 attenuates tumor stemness and evokes antitumor immunity in triple-negative breast cancer by inhibiting IL-6/STAT3 signaling

**DOI:** 10.1186/s13058-023-01642-3

**Published:** 2023-04-17

**Authors:** Heshu Liu, Rui Yan, Zeru Xiao, Xuying Huang, Jiannan Yao, Jian Liu, Guangyu An, Yang Ge

**Affiliations:** Department of Oncology, Beijing Chao-Yang Hospital, Capital Medical University, Beijing, 100020 China

**Keywords:** Triple-negative breast cancer, DCLK1, CSCs, IL-6/STAT3 signaling, The mesenchymal-like subtype

## Abstract

Triple-negative breast cancer (TNBC) exhibits the poorest outcomes among breast cancer subtypes due to the high heterogeneity and a lasting scarcity of effectual treatments. Targeted therapies based on molecular subtypes of TNBC are critical step toward tailoring treatments to improve clinical outcomes. Gastrointestinal cancer stem cell (CSC) marker DCLK1 was reported to be highly expressed in stem cell-rich subtype of TNBC. Here, we firstly explored the impacts of DCLK1 on tumor cells as well as their immune microenvironment in TNBC and potential therapeutic strategies for TNBC patients with high DCLK1 expression. Our results disclosed that DCLK1 overexpression promoted, while knockout of DCLK1 suppressed the CSC-like traits of TNBC cells and resistance to chemotherapeutics. Besides, DCLK1 supported immune escape by inhibiting intratumoral cytotoxic T cell infiltration in TNBC and hence limited immune checkpoint inhibitors efficacy. Mechanistically, bioinformatics analysis revealed that IL-6/STAT3 signaling was significantly enriched in high DCLK1-expressing patients, and our results further revealed that DCLK1 enhanced IL-6 expression and STAT3 activation in TNBC cells, which finally gave rise to upregulated CSC traits and suppressed CD8+ T-cell activity. Inhibiting IL-6/STAT3 pathway by IL-6R antagonist, Tocilizumab or STAT3 inhibitor, S31-201 could abolish DCLK1-promoted malignant phenotypes of TNBC cells. Finally, DCLK1 was identified to be specifically and highly expressed in the mesenchymal-like subtype of TNBC and targeting DCLK1 could improve chemotherapy efficacy and activate antitumor immunity. Overall, our study revealed the potential clinical benefits of targeting DCLK1 in TNBC treatment.

**Supplementary Information:**

The online version contains supplementary material available at 10.1186/s13058-023-01642-3.

## Introduction

Breast cancer is the second most common malignant tumor following lung cancer in women worldwide [1]. Despite advances in therapy, approximately 30% patients were still afflicted by tumor recurrence and distant metastasis [2]. Triple-negative breast cancer (TNBC) is a highly aggressive subtype, characterized by absent expression of estrogen receptor (ER), progesterone receptor (PR) and epidermal growth factor receptor (HER2), which render TNBC lacking effective targets for drug intervention [3], with chemotherapy being the only accessible choice.

TNBC is highly heterogeneous in both biological and clinical aspects, and subtyping is a necessary strategy to categorize TNBC heterogeneity for personalized treatment [3, 4]. Initial classification of TNBC based on bulk mRNA profiles identified 6 molecular subtypes by Brian D. Lehmann et al. in 2011 [5]. With the development of sequencing technology, a more comprehensive analysis based on transcriptomic and genomic data of TNBC patients was performed and four molecular subtypes were classified by Fudan University Shanghai Cancer Center (FUSCC) [6]. No matter how to categorize TNBC, the subtype enriched with stem cells was non-negligible and difficult to treat. CSCs are small population of cells displaying a CD44^+^/CD24^−^ phenotype and high ALDH activity (ALDH+) in breast cancer. Stem-like cells have higher tolerability to chemotherapy and radiotherapy, and they could survive first-line therapy to regenerate the bulk of tumor, hence leading to tumor relapse [7, 8]. Therefore, finding the potential targets to against CSCs to improve outcomes for TNBC patients has been a major challenge in the field.

Doublecortin-like kinase 1 (DCLK1) was originally identified as a microtubule-associated protein (MAP) correlated with neurogenesis and neuronal migration [9]. Recently, DCLK1 has been reported as a CSC marker of gastrointestinal tumor [10–14] and has gained increasing attention. Nakanishi and his colleagues pointed out that DCLK1 was specially expressed on intestinal CSCs rather than normal stem cells, which made DCLK1 a promising target for CSCs suppression [15], and the initiating role of DCLK1 in tumorigenesis was subsequently identified in pancreatic cancer (PC) [10]. Besides, the expression levels of DCLK1 were upregulated in multiple cancers including renal cell carcinoma (RCC) [16], colorectal cancer (CRC) [17] and PC [18]. Our previous study also pointed out that DCLK1 inhibition could restore drug sensitivity of TKI-resistant cancer cells by attenuating tumor cells stemness in lung adenocarcinoma [19]. A recent study demonstrated that DCLK1 was highly expressed in stem cell-rich subtype of TNBC [20], which suggested the close association of DCLK1 with TNBC stemness.

In this study, we found that DCLK1 played a prominent role in maintaining CSC traits, promoting metastasis and reducing infiltration of CD8+ cells in tumor immune microenvironment with a concomitant resistance to chemotherapeutics and immune checkpoint inhibitors (ICIs) in TNBC. The activation of IL-6/STAT3 pathway played an important role in DCLK1-promoting malignant progression in TNBC. Targeting DCLK1-activated IL-6/STAT3 pathway by DCLK1 inhibitor might be a promising strategy to TNBC patients with high DCLK1 expressions.

## Materials and methods

### Cell culture and reagent administration

Human TNBC cell lines including BT549, MDA-MB-468 and human mammary epithelial cells MCF-10A as well as mouse cell line 4T1 were purchased from the Chinese Academy of Medical Sciences (Beijing, China). All these cells were authenticated by STR profiling. The MDA-MB-468 cells were cultured in DMEM medium (BI, China), and the BT549, 4T1 cells were cultured in RPMI-1640 (BI, China) medium. All the mediums were supplemented with 10% FBS (ExCell Bio, FSS500, China) and 1% penicillin/streptomycin (KeyGEN BioTECH, KGY0023, China). DMEM/F12 medium containing 5% HS, 20 ng/ml EGF was prepared for MCF-10A. All cells were cultured with 5% CO2 at 37 °C. Chemotherapy drugs including doxorubicin and cisplatin were from Solarbio (China); IL-6 receptor antagonist, Tocilizumab, was purchased from MCE (USA); STAT3 pathway inhibitor, S31-201, was from Selleck (USA); Recombinant Human IL-6 was from PeproTech (USA).

### Knockout and overexpression of DCLK1

CRISPR/Cas9 technology was used to construct DCLK1-knockout cells as previously described [21]. Briefly, we constructed lentiviral plasmids containing single guide RNA targeting DCLK1 and then transferred the plasmids together with psPAX2 and pMD2G into HEK-293T cells via lipofectamine 3000 (Invitrogen, USA). The control cells were transferred the Lenti-CRISPR V2 plasmid. The lentivirus was harvested at 72 h after transfection and then co-cultured with BT549 cells for three days in the existence of 8 ng/ml polybrene (Beyotime, China). After 3 days of co-culture, we selected out infected cells by 1.5 μg/ml puromycin (Beyotime, China) for 7 days. The single guide RNA (sgRNA) sequences targeting human DCLK1 were as follows: Oligo1-DCLK1-5′-CACCGGAGTAGAGAGCTGACTACCA-3′, Oligo2-DCLK1-5′-AAACTGGTAGTCAGCTCTCTACTCC-3′; sgRNA targeting mouse DCLK1 were as follows: 5′-GCATTTTGATGAGCGGGACA-3′. The overexpressing plasmid containing DCLK1 sequence was purchased from Beijing AUGCT.

### Western blotting

Total proteins of TNBC cells were obtained by the RIPA lysis buffer comprising 1 mM protease inhibitor cocktail and PMSF (Beyotime, China). Then, protein concentrations were detected by the BCA protein assay kit (Invitrogen, USA) and equal amounts of proteins were loaded and separated on a 10% SDS polyacrylamide gel after boiling at 95 °C for 10 min. After electrophoresis, the proteins were electrotransferred onto a 0.22-μm PVDF membrane (Millipore Corporation, USA) at 200 mA for 90 min. Then, the membrane containing proteins was blocked with 8% non-fat milk for 1 h at room temperature (RT) and immunoblotted with the corresponding primary antibodies at 4 °C all night. After washing with TBST, the membranes were hatched with horseradish enzyme-conjugated goat anti-rabbit or mouse (ZSGB-BIO, China) for 1 h and then signals were imaged on the Bio-Rad ChemiDoc MP (Hercules, USA) using chemiluminescence kit (Millipore, USA). The primary antibodies used above were: anti-DCLK1(ab31704, Abcam), anti-SOX2 (3579T, CST), anti-NANOG (4903T, CST), anti-OCT4 (11263-1-AP, Proteintech), anti-CD44 (ab243894, Abcam), anti-BMI (6964T, CST), anti-IL-6 (21865-1-AP, Proteintech), anti-JAK1 (ab133666, Abcam), anti-Phosphor-JAK1 (Y1022 + Y1023) (ab138005, Abcam), anti-STAT3 (10253-2-AP, Proteintech), anti-Phospho-Stat3 (Tyr705) (9145T, CST), anti-ZEB1 (9782T, CST), anti-Vimentin (9782T, CST), anti-E-cadherin (9782T, CST), anti-N-cadherin (9782T, CST), anti-Snail (9782T, CST), anti-GAPDH (abs830030, absin).

### Semiquantitative real-time PCR

The whole-cell mRNA was isolated by Trizol reagent (Sigma, USA). Then, 1 μg extracted RNA was used for reverse transcription to synthesize cDNA (11141ES10, Yeasen, China). qPCR was performed to detect the relative mRNA levels of the genes in different cDNA templates with GAPDH as an internal control. The primer sequences were as follows: GAPDH: forward primer: 5′-AATCCCATCACCATCTTCCA-3′, reverse primer 5′-TGGACTCCACGACGTACTCA-3′; IL-6: forward primer: 5′-ACTCACCTCTTCAGAACGAATTG-3′, reverse primer: 5′-CCATCTTTGGAAGGTTCAGGTTG-3′; DCLK1: forward primer: 5′-CGGTCCACATGCAATAAAAA-3′, reverse primer: 5′-GATATCACCGATGCCATCAAG-3′; OCT4: forward primer: 5′-GGGAGATTGATAACTGGTGTGTT-3′; reverse primer: 5′-GTGTATATCCCAGGGTGATCCTC-3′; SOX2: forward primer: 5′-TGGACAGTTACGCGCACAT-3′; reverse primer: 5′-CGAGTAGGACATGCTGTAGGT-3′; NANOG: forward primer: 5′-AAGGTCCCGGTCAAGAAACAG-3′; reverse primer: 5′-CTTCTGCGTCACACCATTGC-3′.

### Tumorsphere formation assay

This assay was used to evaluate the self-renewal capacity of single TNBC cells, in vitro substitute of stem-like cells [42]. TNBC cells (5000 cells/well) were seeded into a six-well ultralow-attachment plate. All tumorspheres were cultured in DMEM-F12 medium (Gibco, USA), supplemented with 2 mM L-glutamine (Stem cell, USA), 5 µg/ml Insulin (Beyotime, China), 4 mg/ml BSA (Beyotime, China), 6 mg/ml Glucose, 15 ng/mL bFGF (Gibco, USA), and 30 ng/mL recombinant human EGF (Gibco, USA). Tumorspheres were cultured for 1–2 weeks and photographed under microscope fitted with graticule at × 100 magnification. The numbers of spheroids (at least 100 μm diameter) were counted in randomly selected five fields of the six-well ultralow-attachment plate.

### Flow cytometry analysis

CD44^high^/CD24^low^ cells population were considered as stem-like cells in breast cancer [22, 23]. 1 × 10^6^ TNBC cells were harvested and incubated in 200 μl buffer containing anti-human CD44 and anti-human CD24 antibodies for 15 min at RT. After washing with PBS, the labeled cells were detected by flow cytometry. For apoptosis analysis, TNBC cells were collected and incubated in 500 μl solution buffer comprising Annexin V-FITC and PI (KeyGEN BioTECH, China) for 15 min at RT, followed by flow cytometry detection.

### Transwell assay

Transwell assay was used to assess the metastatic potential of TNBC cells. For migration assay, BT549 cells (10 × 10^4^) and MDA-MB-468 cells (5 × 10^4^) were diluted in FBS-free medium and added into the upper space of transwell chambers, which contains 8.0-μm polycarbonate membrane (Corning, 3422, USA). The completed medium containing 10% FBS was added to the lower chamber as chemoattractant. After 16 h of incubation, the migrating cells were fixed with absolute ethanol for 20 min and dyed with crystal violet for 10 min. Then the migrating cells were manually counted under the microscope by selecting 3–4 files/well randomly. Unlike migration assay, invasion assay requires the matrigel (BD Bioscience, USA) to be pre-coated into the upper space in advance.

### In vivo experiments

Adult female BALB/c immunocompromised nude mice and immunocompetent mice (6–8 week) were obtained from Charles River Laboratories (Beijing, China). For subcutaneous tumor growth assays, 1 × 10^6^ 4T1 cells were diluted in 100 μl PBS and injected subcutaneously on the abdomen of each mouse (n = 6 per group). Tumor sizes were measured every 3–5 days and then the following method was used to calculate tumor volumes: Volume = (Length × Width^2^)/2. Finally, the mice were sacrificed and the subcutaneous tumors were photographed and harvested for immunohistochemistry (IHC) staining analysis. For lung metastatic mouse model, 2 × 10^6^ cells in 100 μl PBS were intravenously into the tail vein of each mouse (n = 4 per group). After 7–8 weeks, the lung tissues were harvested to count metastatic lung nodules and H&E staining.

### Flow cytometry analysis of tumor-infiltrating lymphocytes

To prepare single-cell suspensions, tumor tissues were minced and digested with 2 mg/ml collagenase type I (Worthington Biochemical, USA) for 1 h at 37 °C followed by filtration with 200-mesh gauze. The acquired single-cell suspensions were incubated at RT for 15 min with fluorescence-labeled antibodies against CD45, CD3, CD4, CD8, IFN-γ. For intracellular cytokine staining, the Intracellular Fixation and Permeabilization kit (Invitrogen, USA) was used according to the manufacturer's instructions. After staining, single-cell suspensions were detected by flow cytometry. The fluorescence-labeled antibodies used above were: APC anti-mouse CD45 Antibody (1:500, Biolegend, 103111), Brilliant Violet 421 anti-mouse CD3 Antibody (1:500, Biolegend, 100227), FITC anti-mouse CD4 (1:500, Biolegend, 100406), PE anti-mouse CD8a (1:500, Biolegend, 100708), 7-AAD Viability Staining Solution (1:500, BD, 559925).

### Immunohistochemistry

The harvested subcutaneous tumor tissues and metastatic lung tissues from above in vivo experiments were fixed with neutral formalin fixative (Solarbio, China) for 48 h and embedded in paraffin for IHC analysis. Briefly, 5-μm-thick sections from above samples were deparaffinized in xylene and hydrated in a descending alcohol series. After antigen retrieval with EDTA, the sections were blocked with goat serum for 1 h (ZSGB-BIO, China) followed by incubation with the corresponding primary antibodies 37 °C overnight. After incubation with the secondary antibody (ZSGB-BIO, China), the 3, 3′-diaminobenzidine (DAB, ZSGB-BIO, China) was added for IHC signaling detection. The following antibodies were used: anti-DCLK1 (, ab31704, Abcam), anti-IL-6 (Proteintech, 21865-1-AP), anti-Phospho-Stat3 (Tyr705) (CST, 9145 T), anti-CD8 (ab217344, Abcam).

### CD8 deletion experiment

To delete CD8+ T cells, mice were injected intraperitoneally with anti-CD8α antibody (BioXcell, BE0117, USA) at a dose of 10 mg/kg, one day before the subcutaneous injection of 4T1 cells and the anti-CD8α antibody were injected with the same dose every 5 days thereafter. To assess the delete efficiency of CD8+ T cells, T cells from single-cell suspensions of spleens, lymph nodes and thymus were analyzed by flow cytometry.

### Drug sensitivity measurements

Chemotherapy drugs used in this study were doxorubicin and cisplatin, and IC50 values were used to assess drug sensitivity. TNBC cells were seeded in a 96-well plate at a density of 5000 cells/well. Next day, doxorubicin or cisplatin were added to the cell culture medium at fold-increasing concentrations and after 24 h, Cell Counting Kit-8 (CCK8, Yeasen, China) was used to detect cell viability for further IC50 analysis.

### Bioinformatics analysis

The TNBC RNA sequencing data of FUSCC were downloaded from The National Omics Data (http://www.biosino.org/node). The kmplot was used to analyze the effects of DCLK1 and IL-6 on prognosis of BC patients (https://kmplot.com/). The Tumor Immune Dysfunction and Exclusion (TIDE, http://tide.dfci.harvard.edu.) analysis was used to evaluate the effects of DCLK1 and IL-6 expressions on the responsiveness of ICIs (anti-PD-1 and anti-CTLA4).

### Statistical analyses

Experimental data were analyzed by GraphPad Prism 7 and presented as mean ± SD unless otherwise specified. The student's t test was used for parametric analyses of two groups, and the one-way analysis of variance was used for multi-group comparisons in GraphPad Prism. And R software (version 3.6.3) was used for Chi-squared test and Kruskal–Wallis analysis. Spearman correlation analysis was used for the correlation between DCLK1 and IL-6 expression. All independent experiments were repeated at least in triplicate. P values < 0.05 were considered statistically significant (*p < 0.05, **p < 0.01, ***p < 0.001, ****p < 0.0001).

## Results

### DCLK1 promotes the CSC traits of TNBC cells

**Fig. 1:**
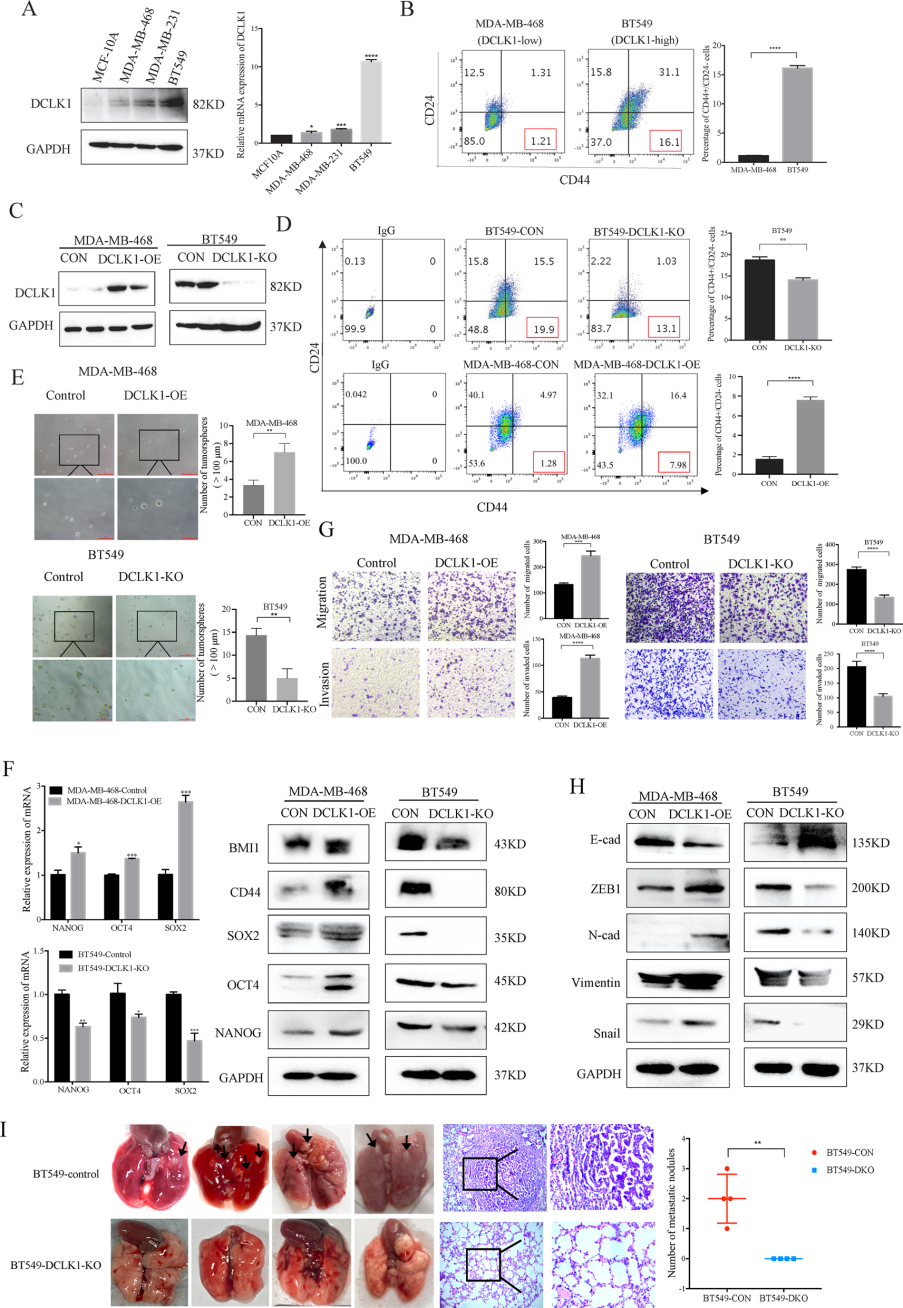
DCLK1 promotes CSC-like properties of TNBC cells. **a** The protein and mRNA levels of DCLK1 were higher in TNBC cells than normal breast epithelial cells MCF10A. **b** Flow cytometry analysis revealing the percentage of stem-like subpopulation CD44^high^/CD24^low^ in BT549 and MDA-MB-468 cells. **c** Western blotting analysis of DCLK1 expression by DCLK1 knockout in BT549 cells and DCLK1 overexpression in MDA-MB-468 cells. **d** Flow cytometry analyzing the percentage of stem-like subpopulation CD44^high^/CD24^low^ after DCLK1 knockout in BT549 cells (upper) and DCLK1 overexpression in MDA-MB-468 cells (lower). **e** Tumorsphere formation assays analyzing the impacts of DCLK1 knockout and DCLK1 overexpression on self-renewal capabilities of TNBC cells. Scale bars: 1000 μm. **f** Real-time qPCR (left) and Western blotting (right) analysis of CSC-associated markers in DCLK1-overexpressing MDA-MB-468 cells and DCLK1-knockout BT549 cells. **g** Transwell assays showing the enhanced migratory/invasive abilities in DCLK1-overexpressing MDA-MB-468 cells (left) as well as the inhibited migratory/invasive abilities in DCLK1-knockout BT549 cells compared to the corresponding control cells (right). **h** Western blotting determination of the effects of DCLK1 expression on EMT-associated markers. **i** Lung metastases in the BALB/c nude mice intravenously injected with DCLK1-knockout BT549 cells and the control cells (left), comparison of lung metastases by H&E staining (middle) and counting the number of metastases (right)

DCLK1 plays a significant role in maintaining CSC-like properties [10, 24]. In TNBC, the treatment for CSC-enriched subtype remains a major clinical challenge due to the absence of effective targets. To investigate the regulatory functions of DCLK1 in TNBC, we performed the following studies. Firstly, we detected the basic expression of DCLK1 in TNBC cells and normal human mammary epithelial cells, the results showed that both mRNA and protein levels of DCLK1 were significantly higher in TNBC cell lines compared to normal human mammary epithelial cells MCF-10A (Fig. 1a). The percentage of CD44^high^/CD24^low^ stem-like subpopulation was also higher in BT549 cells than MDA-MB-468 cells, which was consistent with the expression levels of DCLK1 in TNBC cells (Fig. 1b). To explore its regulatory roles in TNBC, DCLK1 was knocked out in BT549 cells and overexpressed in MDA-MB-468 cells using lentiviruses transduction (Fig. 1c). We found that the percentage of CD44^high^/CD24^low^ subpopulation in BT549 cells was significantly decreased after DCLK 1 knockout (Fig. 1d) while DCLK1 overexpression in MDA-MB-468 cells increased the percentage of CD44^high^/CD24^low^ subpopulation. Mammosphere formation assays were used to assess the effects of DCLK1 on CSC self-renewal in vitro. We found that DCLK1 overexpression significantly promoted the numbers and volume of mammospheres in MDA-MB-468 cells while DCLK1 knockout in BT549 cells inhibited (Fig. 1e). CSC-transcription factors (TFs) including SOX2, OCT4, NANOG play an indispensable role in maintaining self-renewal and pluripotency of CSCs. Our results demonstrated that DCLK1 promoted the expression of CSC-associated TFs at both mRNA and protein levels (Fig. 1f). CSC-like properties are highly correlated to metastasis and tumor recurrence [25]. To determine whether DCLK1 regulates the invasive behaviors of TNBC cells, transwell assays were performed. As anticipated, DCLK1 overexpression significantly enhanced the migratory/invasive abilities of TNBC cells while DCLK1 knockout suppressed (Fig. 1g). Moreover, the epithelial marker E-cad was decreased and the mesenchymal markers including N-cad, ZEB1, Snail, Vimentin were increased in DCLK1-overexpressing cells and the opposite phenotypes were determined by DCLK1 knockout in BT549 cells (Fig. 1h). The impact of DCLK1 on invasive potential in vivo was verified by a xenograft metastasis model. The BT549-DCLK-KO cells and the BT549-control cells were injected into the tail vein of nude mice for the establishment of lung metastasis model. As shown in Fig. 1i, more lung metastases were observed in the group injected with BT549-control cells, while no visible metastases in the DCLK1-knockout group, which were further verified by H&E staining (Fig. 1i, middle). All these data revealed the critical roles of DCLK1 in promoting CSC-like traits in TNBC.

### DCLK1-enhanced stemness increases the tolerance of TNBC cells to chemotherapy

**Fig. 2:**
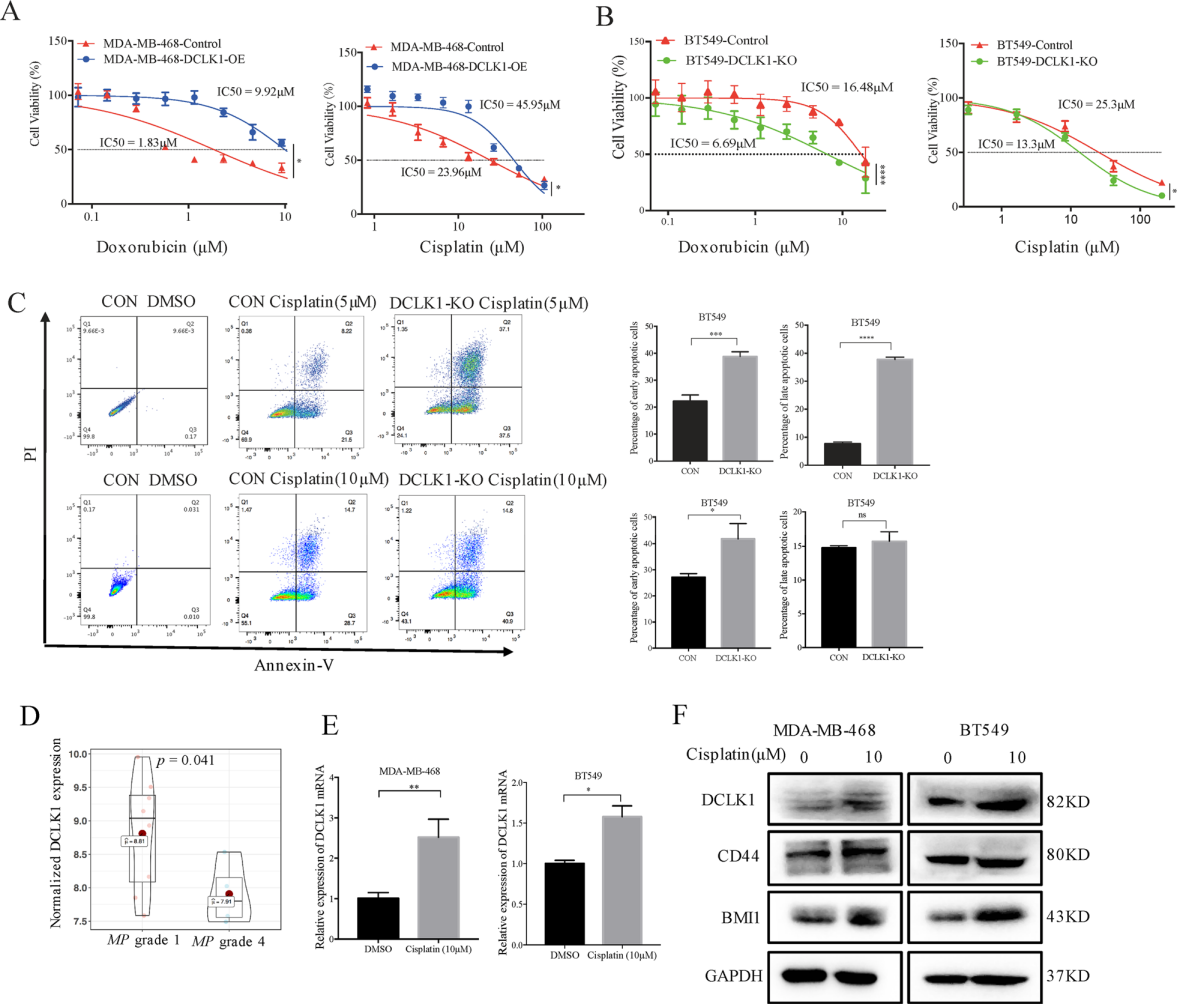
DCLK1 reduces the sensitivity of TNBC cells to chemotherapeutics. **a** CCK8 assays showing a higher nanomolar IC50 by doxorubicin (left) and cisplatin (right) in DCLK1-overexperssing cells than the corresponding control (doxorubicin, 1.83–9.92 μM; cisplatin, 23.96–45.95 μM). **b** CCK8 assays showing a lower nanomolar IC50 by doxorubicin (left) and cisplatin (right) in DCLK1-knockout cells than the corresponding control (doxorubicin, 16.48–6.69 μM; cisplatin, 25.3–13.3 μM). **c** Annexin V analysis was used to detect the effects of cisplatin on apoptosis of BT549-control cells and BT549-DCLK1-KO cells. **d** GSE103668 dataset analysis of the association between DCLK1 expressions and the efficacy of neoadjuvant chemotherapy in TNBC. **e** qPCR analysis showing the increased expression of DCLK1 in TNBC cells after cisplatin treatment. **f** Western blotting disclosing the increased expression of CSC-related markers in TNBC cells after cisplatin treatment

CSCs are important causes of tumor chemotherapy resistance [26, 27]. The prominent roles of DCLK1 in maintaining stemness of TNBC cells suggested that DCLK1 might be the critical factor causing the insensitivity to chemotherapy. Therefore, we further explored the association between DCLK1 and the sensitivity of TNBC cells to chemotherapy drugs. Cisplatin and doxorubicin, the two most common chemotherapeutics for TNBC, were selected as representative drugs. The drug sensitivity assays showed that DCLK1 overexpression decreased the sensitivity of MDA-MB-468 cells to doxorubicin and cisplatin, as demonstrated by the increased values of IC50 (doxorubicin, 1.83–9.92 μM; cisplatin, 23.96–45.95 μM) compared to the control cells (Fig. 2a). Moreover, the knockout of DCLK1 in BT549 cells increased the drug sensitivity, demonstrated by the decreased values of IC50 (doxorubicin, 16.48–6.69 μM; cisplatin, 25.3–13.3 μM) (Fig. 2b). Annexin-V/PI flow analysis results also showed that DCLK1 knockout significantly increased the percentage of apoptotic cells induced by cisplatin (Fig. 2c). Then, the GEO dataset (GSE103668) including 21 TNBC patients treated with cisplatin & bevacizumab before surgery were analyzed and results revealed that patients with no significant reduction in tumor size, also ranked as Miller-Payne (MP) grade 1 according to Miller-Payne response score tended to express higher DCLK1 levels than patients with tumor reduction of more than 90%, also known as MP grade 4 (Fig. 2d). qPCR analysis showed that TNBC cells surviving cisplatin treatment expressed higher DCLK1 than the corresponding parental cells (Fig. 2e). Western blotting further verified that these TNBC cells surviving cisplatin treatment exhibited enhanced CSC-like properties, as demonstrated by higher expression of DCLK1, CD44 and BMI1 (Fig. 2f). All these data revealed the importance of targeting DCLK1 in improving chemotherapy efficacy in TNBC.

### DCLK1 inhibits antitumor immunity and ICIs efficacy by reducing CD8+ T cells infiltration

**Fig. 3:**
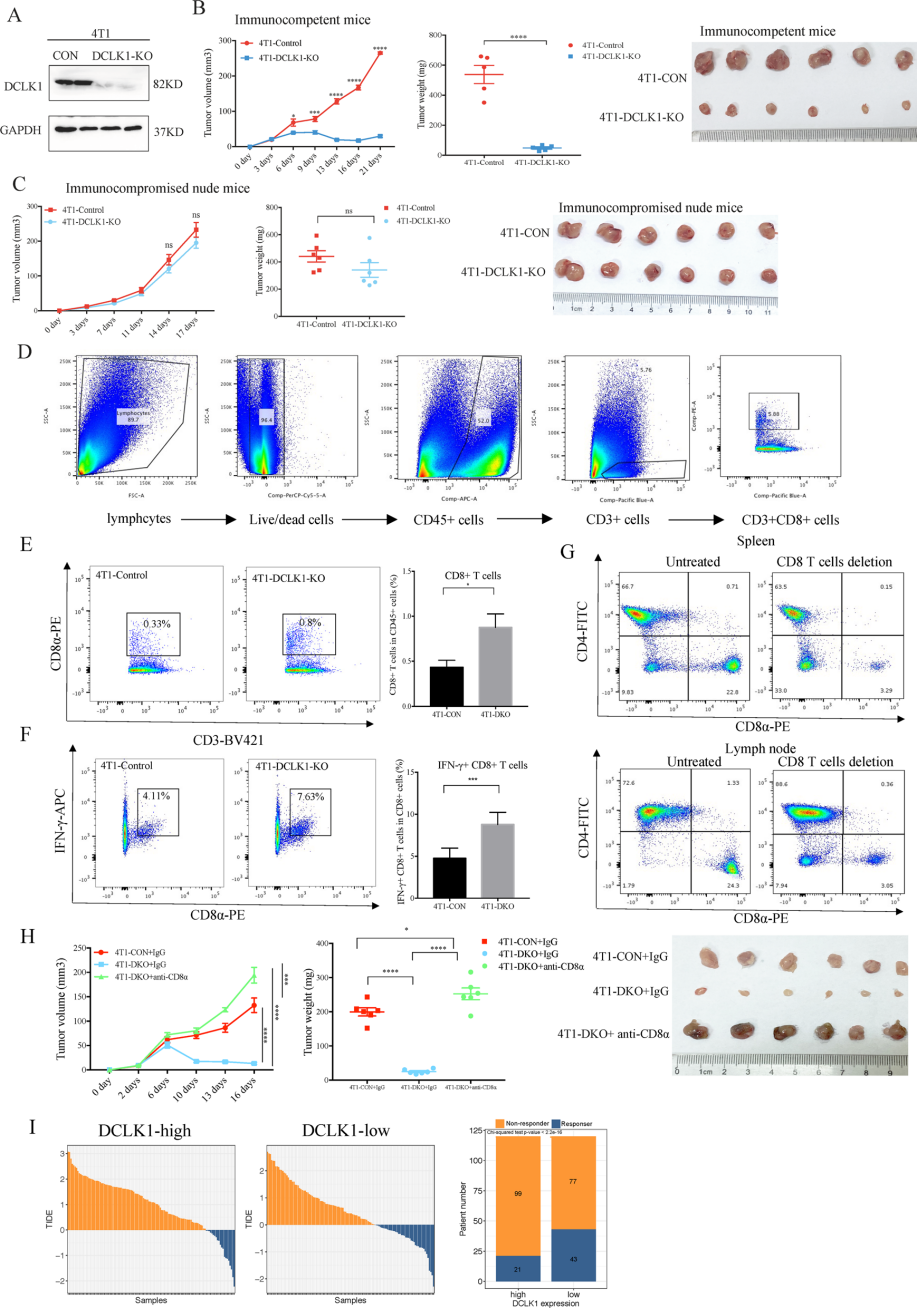
DCLK1 inhibits the infiltration of CD8+ T cells and limits the ICIs efficacy. **a** Western blotting verifying the knockout of DCLK1 in 4T1 cells. **b, c** Immunocompetent BALB/c mice (**b**) and immunocompromised BALB/c nude mice (**c**) were inoculated with DCLK1-knockout 4T1 cells and the control cells. Tumor growth (left), tumor weight (middle) and size (right) were measured. **d **The representative images of the gating strategy used to define CD8+ T cells in mouse tumors. **e, f** Flow cytometry analyzing the percentage of CD8+ T cells (**e**) and IFN-γ+ CD8+ T cells (**f**) in subcutaneous tumor tissues from immunocompetent mouse 4T1 model. **g** Flow cytometry analysis of the proportion of CD8+ T cells in the spleen (upper) and peripheral lymph nodes (lower) after anti-CD8α treatment. **h** Tumor growth curve (left), tumor weight (middle) and tumor size (right) of immunocompetent 4T1 subcutaneous tumors treated as indicated. (n = 6 per group). **i** TIDE analysis showing a lower response in TNBC patients with a higher DCLK1 expression (left, 18%, 21/120) than patients with a lower DCLK1 expression (right, 36%, 42/120) in the FUSCC dataset

DCLK1 has been reported to be associated with tumorigenic immune infiltrates in gastrointestinal tumor and renal cell carcinoma [28, 29]. A better understanding of the crosstalk between CSCs and the immune microenvironment might be the key to improve the efficacy of immunotherapy and CSC-targeted therapy in TNBC [3, 30]. Therefore, we investigated the influence of DCLK1 on anti-tumor immunity by using 4T1 syngeneic mouse model. CRISPR-Cas9 mediated DCLK1 knockout in 4T1 cells was verified by Western blotting (Fig. 3a). The 4T1-control cells and 4T1-DCLK1-knockout cells were subcutaneously injected into the abdomen of immunocompetent (Fig. 3b) and immunocompromised mice (Fig. 3c). As the results showed, DCLK1 knockout resulted in a significant inhibitory effect on tumor growth in immunocompetent BALB/c mice (Fig. 3b), while in immunocompromised BALB/c nude mice, the difference between DCLK1-knockout tumors and the control group was not significant (Fig. 3c). The results demonstrated that the effects of DCLK1 knockout on tumor growth mainly depend upon anti-tumor immune response. Therefore, we detected the infiltration of CD8+ T cells and IFN+ CD8+ T cells in tumors by flow cytometry. The representative images of the gating strategy used to define CD8+ T cells in mouse tumors are shown in Fig. 3d. The results disclosed the increases in the proportion of both CD8+ T cells and IFN+ CD8+ T cells in tumors with DCLK1 knockout (Fig. 3e, f). To further assess whether the increased infiltration of CD8+ T cells contributed to antitumor immunity of DCLK1 knockout, we deleted CD8+ T cells by anti-CD8α treatment and the deletion efficiency were identified by analysis of T cells in spleen, thymus and lymph nodes of mice treated with anti-CD8α antibody. As the results showed (Fig. 3g), the percentage of CD8+ T cell in spleens and lymph nodes were significantly decreased and not significantly changed in the thymus (Additional file 1: Figure S1). Anti-CD8 treatment significantly abrogated the anti-tumor effects of DCLK1 knockout (Fig. 3h), suggesting antitumor immunity of DCLK1 knockout relied on CD8+ T cells. Considering that the efficacy ICIs requires CD8+ T cells infiltrations in tumor, we performed TIDE analysis based on FUSCC dataset to predict the correlation between DCLK1 expression levels and the responsiveness of ICIs in TNBC patients. As the results displayed in Fig. 3i, patients with higher DCLK1 expressions (18%, 21/120) exhibited lower responsiveness to ICIs than patients with lower expressions (36%, 43/120), primarily because DCLK1 induced immune escape by reducing the CD8+ T cells infiltration. All these data revealed the importance of targeting DCLK1 in evoking anti-tumor immunity.

### DCLK1 activates IL-6/STAT3 pathway in TNBC

**Fig. 4:**
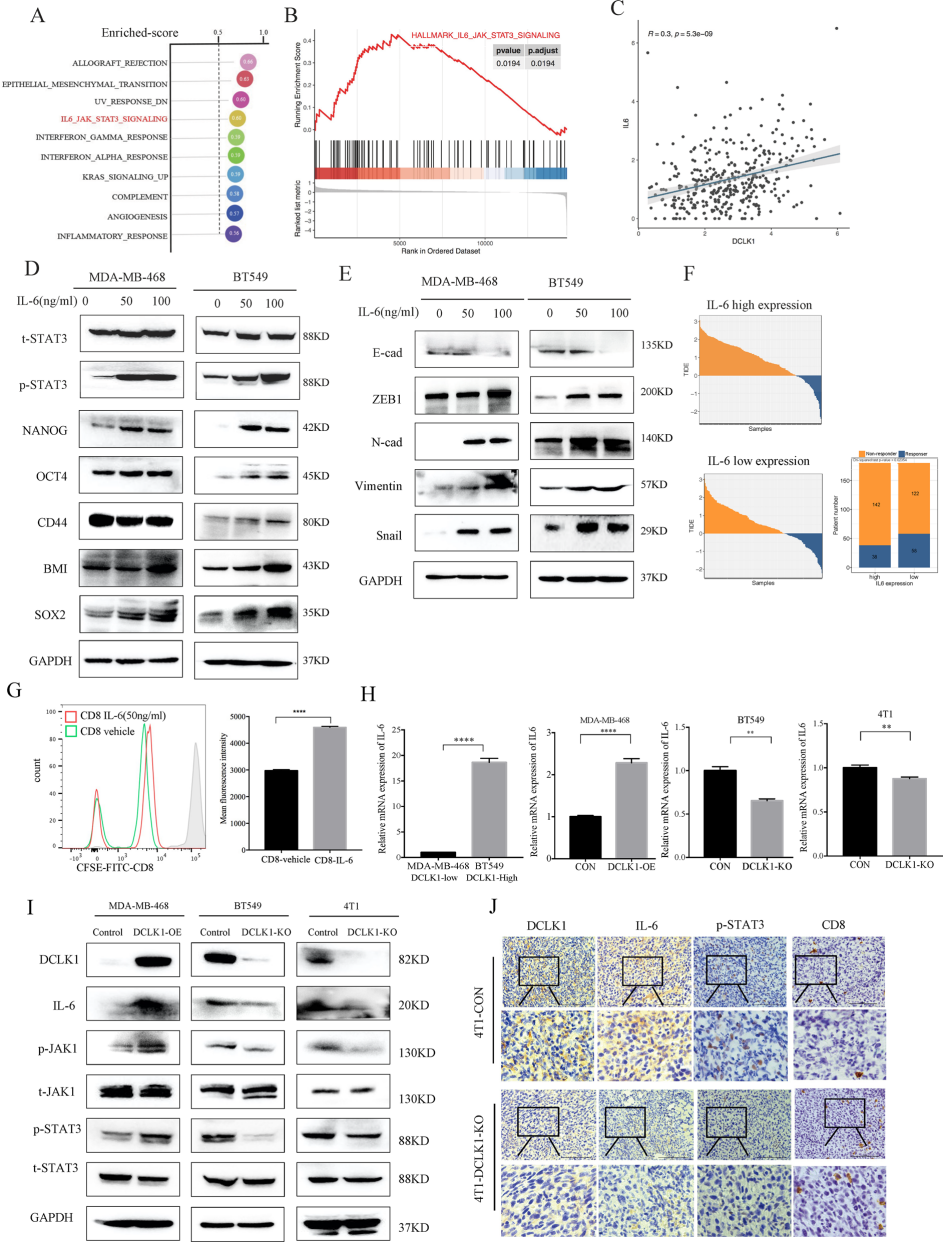
DCLK1 activates IL-6/JAK1/STAT3 signaling. **a** Gene set variation analysis (GSVA) showing DCLK1-associated pathways in TNBC. **b** Gene set enrichment analysis (GSEA) revealing the significant activation of IL-6/JAK/STAT3 pathway in DCLK1-high expressed TNBC patients. **c** Spearman correlation analysis showing the positive association between DCLK1 expression and IL6 expression. **d, e** Western blotting to determine expression levels of phosphorylated STAT3 (Tyr705) and total STAT3 as well as CSC-associated markers (**d**) and EMT-associated markers (**e**) in TNBC cells treated with exogenous IL-6 stimulation for 24 h. **f** TIDE analysis showing a lower response in TNBC patients with a higher IL-6 expression (left, 21%, 38/180) than patients with a lower IL-6 expression (right, 32%, 58/122) in the FUSCC dataset. **g** CFSE-based T-cell proliferation assay revealing a lower activity of CD8+ T cell treated with exogenous IL-6 stimulation for 72 h compared to the control group. **h** Real-time qPCR determination of the effects of DCLK1 on IL-6 mRNA expression levels. **i** Western blotting detection of activated IL-6/JAK1/STAT3 signaling in DCLK1-overexpressing MDA-MB-468 cells (left) and inactivated IL-6/JAK1/STAT3 signaling in DCLK1-knockout BT549 cells (middle), 4T1 cell (right). **j** IHC examination of DCLK1, IL-6, phosphorylated STAT3 (Tyr705) and CD8 in the control and DCLK1-knockout 4T1 subcutaneous tumors

To explore the potential mechanism of DCLK1-mediated malignant behaviors in TNBC, Gene Set Variation Analysis (GSVA) analysis was performed and the result showed that IL-6/JAK/STAT3 pathway was hyper-activated in TNBC patients with high DCLK1 expression (Enriched-score = 0.6, *p* < 0.0001, Fig. 4a) which was further confirmed by Gene Set Enrichment Analysis (GSEA) analysis (*p* = 0.0194, Fig. 4b). Spearman correlation analysis disclosed that DCLK1 expressions were positively correlated with IL-6 expressions (Spearman *r* = 0.3, *p* < 0.0001, Fig. 4c). Recent studies reported that the IL-6/STAT3 signaling acted to drive proliferation, invasiveness, and metastasis of cancer cells [31, 32], while strongly inhibited anti-tumor immunity [33, 34]. To verify the functions of IL-6/STAT3 pathway in TNBC, we treated TNBC cells with exogenous recombinant human IL-6 for 24 h, then detected the changes in stem-like properties. The results showed that upregulated STAT3 signaling by IL-6 stimulation enhanced the CSC-related characteristics of TNBC cells, including the upregulated CSC-markers (Fig. 4d) as well as increased percentages of CD44^high^/CD24^low^ populations (Additional file 2: Figure S2A). Besides, EMT-associated markers and migratory/invasive abilities of TNBC cells treated with IL-6 stimulation were also enhanced (Fig. 4e, Additional file 2: Fig. S2B). In addition to direct impacts on tumor cells, IL-6 and downstream JAK/STAT3 pathway also have profound effects on tumor immunosuppressive environment. Subsequently, we performed TIDE analysis to evaluate the effects of IL-6 on ICIs responsiveness in TNBC patients. As anticipated, patients with higher IL-6 expression (21%, 38/180) were predicted a better response to ICIs compared to those with lower IL-6 expression (32%, 58/180) (Fig. 4f). To investigate whether IL-6 has a direct effect on CD8+ T-cell function, we isolated peripheral blood CD8+ T cells from healthy donors and stimulated with exogenous IL-6 for 72 h. Then CFSE-based proliferative assay was performed and results showed the CD8+ T cell-activity was significantly inhibited compare to those untreated with exogenous IL-6 (Fig. 4g). These data revealed the crucial roles of IL-6/STAT3 signaling in promoting CSC-traits of TNBC cells and inhibiting anti-tumor immunity, which were consistent with the functions of DCLK1 in TNBC. Therefore, we further explored whether DCLK1 functioned by activating IL-6/STAT3 signaling. Consistent with above analysis, we found that IL-6 expression levels were significantly higher in BT549 cells than MDA-MB-468 cells, which were consistent with DCLK1 basic expression pattern, and DCLK1 overexpression promoted IL-6 mRNA expression while DCLK1 knockout inhibited (Fig. 4h). Then Western blotting analysis revealed that DCLK1 overexpression significantly increased IL-6 protein levels as well as the phosphorylation levels of JAK1 at Y1022 + Y1023 and STAT3 at Tyr705 in DCLK1-overexpressing MDA-MB-468 cells compared to the control cells, while the knockout of DCLK1 in BT549 and 4T1 cells showed opposite effects (Fig. 4i). In order to clarify whether DCLK1-mediated IL-6/STAT3 pathway activation exists in vivo, we tested the expression of IL-6 and phospho-STAT3 in obtained 4T1 subcutaneous tumors by IHC, the results showed that IL-6 and phospho-STAT3 expression levels were decreased in 4T1-knockout tumors, while CD8+ T cells were increased (Fig. 4j). All these data supported that DCLK1 significantly activated IL-6/STAT3 pathway in TNBC both in vitro and in vivo.

### DCLK1 promotes TNBC progression by activating IL-6/STAT3 signaling

**Fig. 5:**
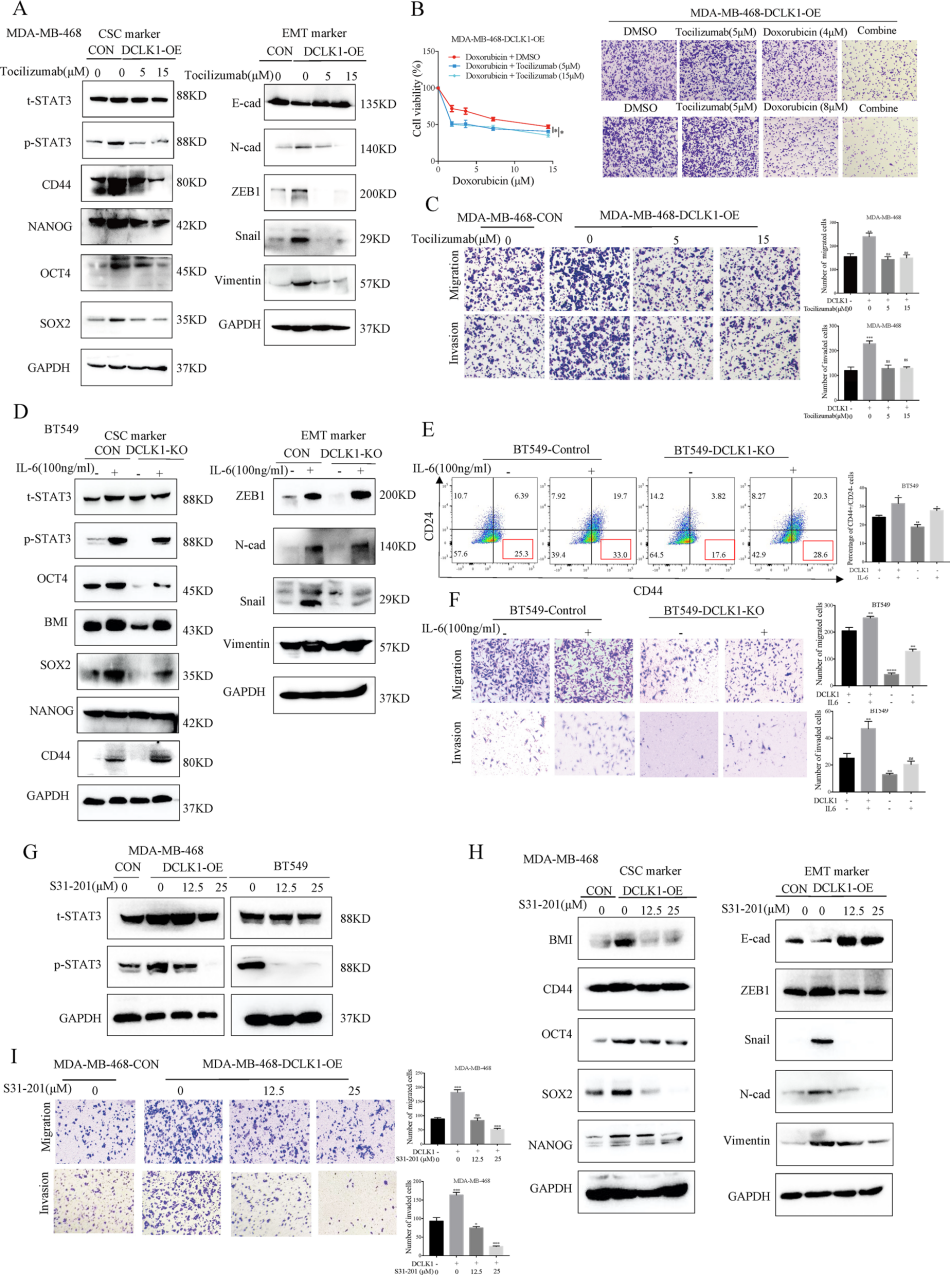
DCLK1 promotes TNBC progression by activating IL6/STAT3 signaling. **a** Western blotting showing the decreased expression levels of phosphorylated STAT3 (Tyr705) as well as CSC-associated markers (left) and EMT-associated markers (right) in DCLK1-overexpressing MDA-MB-468 cells treated with IL-6R antagonist Tocilizumab for 24 h. **b** CCK8 assays to determine the sensitivity of MDA-MB-468-DCLK1-OE cells to doxorubicin after treating with Tocilizumab or DMSO (left), and the cells surviving different treatments were also showed by crystal violet (right). **c** Transwell assays to compare the migratory/invasive abilities of MDA-MB-468 cells with different treatment as indicated. **d** Western blotting showing the expression levels of phosphorylated STAT3 (Tyr705) as well as CSC-associated markers (left) and EMT-associated markers (right) in BT549 cells treated as indicated. **e** Flow cytometry analyzing the percentage of stem-like subpopulation CD44^high^/CD24^low^ in BT549 cells treated as indicated. **f** Transwell assays to compare the migratory/invasive abilities of BT549 cells with different treatment as indicated. **g** Western blotting to detect the inhibitory effects of STAT3 inhibitor S31-201 to TNBC cells. H-I. Western blotting (**h**) and transwell assays (**i**) to determine the inhibitory effects of S31-201 to DCLK1-enhanced CSC traits in MDA-MB-468 cells

Based on above findings, we hypothesized that IL-6/STAT3 signaling played indispensable roles in DCLK1-mediated progression of TNBC. We firstly treated DCLK1-overexpressing MDA-MB-468 cells with IL-6R antagonist, Tocilizumab for 24 h at 5 μM and 15 μM, respectively. We found that Tocilizumab dramatically attenuated the DCLK1-induced STAT3 activation, as well as the expressions of CSC and EMT-related markers (Fig. 5a). Besides, DCLK1-induced resistance to chemotherapeutics in MDA-MB-468-DCLK1-OE cells were also inhibited (Fig. 5b), and transwell assays also showed the migratory/invasive abilities of DCLK1-overexpressing cells were inhibited after Tocilizumab treatments (Fig. 5c). Moreover, a rescue methodology by exogenous IL-6 was applied in DCLK1-knockout BT549 cells. As results shown in Fig. 5d, IL-6 could restore phosphorylation level of STAT3 and expression levels of CSC-related markers in DCLK1-knockout BT549 cells. Moreover, the percentage of CD44^high^/CD24^low^ subpopulation (Fig. 5e) and the invasive potential (Fig. 5f) were also rescued in BT549-DCLK1-KO cells after IL-6 treatment. To further determine that STAT3 activation was the key downstream effector of DCLK1-activated IL-6 signaling, a pharmacological inhibitor of STAT3 phosphorylation, S3I-201, was used in BT549 cells and DCLK1-overexpressing MDA-MB-468 cells and the phosphorylation levels of STAT3 were inhibited at 12.5 μM and 25 μM (Fig. 5g). Besides, DCLK1-induced CSC traits were significantly suppressed by S31-201, in a dose-dependent manner (12.5 μM, 25 μM), as demonstrated by the downregulated CSC and EMT-associated markers (Fig. 5h), and the corresponding migratory/invasive abilities were also inhibited in DCLK1-overexpressing MDA-MB-468 cells treated with inhibitor S31-201 (Fig. 5i). All these data disclosed that DCLK1-promoed TNBC progression via IL-6/STAT3 pathway.

### DCLK1 is a promising target in mesenchymal-like subtype of TNBC

**Fig. 6:**
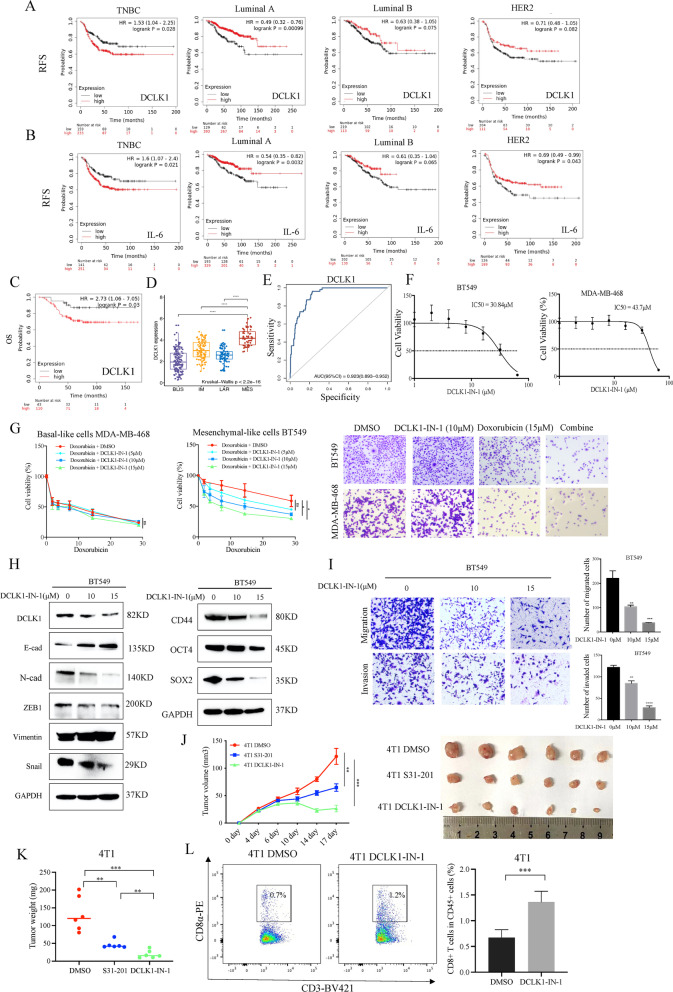
DCLK1 is a promising target in mesenchymal-like subtype of TNBC. **a, b** High mRNA expression of DCLK1 (**a**) and IL-6 (**b**) predicts reduced recurrence-free survival (RFS) in TNBC (n = 392) subtype rather than Luminal A (n = 522), Luminal B (n = 332) or HER2 (n = 315) subtype according to KM-potter database. **c** High mRNA expression of DCLK1 and predicts reduced overall survival (OS) in TNBC tissues according to KM-plotter database (n = 153). **d** The boxplot showing a significantly higher expression of DCLK1 in the MES subtype than the other three subtypes. **e** Receiver operating characteristic (ROC) curve for using DCLK1 expression to identify the MES subtype. **f** CCK8 assays to determine the toxicity of DCLK1 inhibitor, DCLK1-IN-1, on TNBC cells. **g** CCK8 assays to determine the cell viability of mesenchymal-like cells BT549 and basal-like cells MDA-MB-468 with different treatment as indicated (left and middle), and the cells surviving different treatments were also showed by crystal violet (right). **h** Western blotting showing the effects of DCLK1-IN-1 on EMT and CSC-associated marker in BT549 cells. **i** Transwell assays showing the inhibited migratory/invasive abilities in BT549 cells treated with DCLK1-IN-1. **j** Tumor growth curves showing the anti-tumor effects after DCLK1-IN-1 or S31-201 treatment (left), tumors from the indicated treatments were removed and photographed (right). (n = 6 per group). **k** Excised tumor weights showing a significant decrease after DCLK1-IN-1 or S31-201 treatment. **l** Flow cytometry analyzing the percentage of CD8+ T cells in subcutaneous tumor tissues after the indicated treatments

To determine whether DCLK1 and IL-6 were associated with the prognosis of TNBC patients, the survival curves based on GEO datasets were performed by Kaplan–Meier Plotter (https://kmplot.com/analysis/). We found that high DCLK1 and IL-6 expression predicted shorter relapse-free survival (RFS) in TNBC (n = 392) but not in luminal A (n = 522), luminal B (n = 332) and HER2 (n = 315) subtypes, which suggested the dominant roles of DCLK1 and IL-6 in TNBC tumor recurrence (Fig. 6a, b). In addition, higher DCLK1 expression in TNBC also predicted worse overall survival (n = 153) (Fig. 6c). Among TNBC subtypes, a subtype named mesenchymal-like (MES) subtype is characterized by the abundance of CSCs and CSC-related signaling pathways. Notably, our results showed that DCLK1 was highly expressed in the MES subtype (Fig. 6d), which was consistent with the previous study from Shen Zhao et al. [20]. Subsequently, we also performed receiver operating characteristic (ROC) analysis and confirmed the predictive efficacy of DCLK1 in discriminating MES subtype of TNBC patients (Fig. 6e, AUC = 0.923, 95% CI: 0.893–0.952). Considering the important roles of DCLK1 in TNBC progression, we supposed that DCLK1 might be a promising target for the MES subtype and DCLK1 specific inhibitor, DCLK1-IN-1, was applied for the subsequent investigations. The cytotoxicity of DCLK1 on TNBC cells was evaluated firstly by CCK-8 assays, and the results are shown in Fig. 6f. Then, we found that DCLK1-IN-1 combined with chemotherapeutic doxorubicin could effectively and synergistically kill mesenchymal-like cells BT549, while this coordination of DCLK1-IN-1 and chemotherapeutics was not significant in the basal-like cells MDA-MB-468 (Fig. 6g), which might due to the inherent sensitivity of basal-like subtype to chemotherapy and the low DCLK1 expression [5]. Moreover, the CSC and EMT-related markers (Fig. 6h) as well as migratory/invasive potential (Fig. 6i) were also inhibited by DCLK1-IN-1 in BT549 cells. To investigate the effect of DCLK1-IN-1 on antitumor immunity, we conducted in vivo experiments using 4T1 subcutaneous tumor model. 4T1 cells were subcutaneously transplanted into the abdomen of immunocompetent BALB/c mice. Four days post-implantation, these tumor-bearing mice were randomly divided into three groups and treated with DCLK1-IN-1 via oral gavage (20 mg/kg, every other day), S31-201 intraperitoneally (5 mg/kg, three times a week) and the corresponding solvent, respectively. The tumor growth curves showed that DCLK1-IN-1 and S31-201 significantly inhibited the tumor growth when compared to the solvent group, especially DCLK1-IN-1 (Fig. 6j). The average weight of tumor tissues treated by DCLK1-IN-1 or S31-201 was also lower (Fig. 6k). To investigate whether the anti-tumor effects depend on antitumor immunity, we detected the infiltration of CD8+ T cells in tumor tissues treated with DCLK1-IN-1 and the corresponding solvent. As the results displayed in Fig. 6l, the proportion of CD8+ T cells were significantly increased in subcutaneous tumors treated with DCLK1-IN-1. All these data identified the mesenchymal-like subtype of TNBC as most likely to benefit from the DCLK1-targeting treatment.

### Schematic illustration

**Fig. 7:**
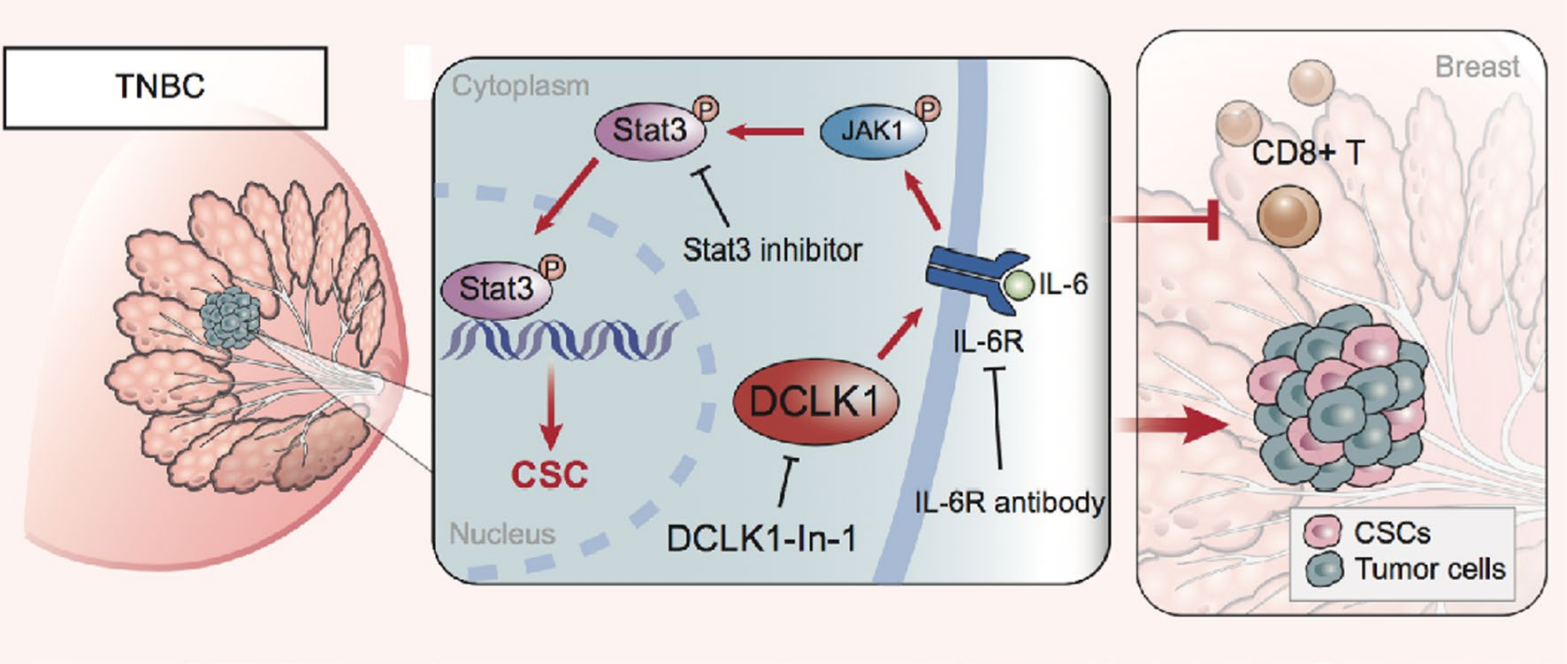
DCLK1-activated IL-6/STAT3 pathway plays critical roles in CSC-like properties and anti-tumor immunity in TNBC. DCLK1 upregulates IL-6 expression and activates JAK1/STAT3 signaling, which promotes tumor cell stemness and inhibits CD8+ T cell-mediated antitumor immunity. DCLK1-IN-1 may be a promising treatment for TNBC patients with high DCLK1 expressions

Our study revealed the important roles of DCLK1 in promoting CSC-like properties and inhibiting the function of CD8+ T cells, which was mainly depending on the activation of IL-6/JAK1/STAT3 signaling (Fig. 7). Targeting DCLK1 with DCLK1-IN-1 might be a promising treatment by increasing cellular sensitivity to chemotherapeutics (Fig. 2) and activating the antitumor immunity (Figs. 3, 6). The specific high expression of DCLK1 in the MES subtype further pointed out the potential clinical application value of DCLK1, which is significant for individualized treatment based on TNBC subtypes.

## Discussion

TNBC is a wide compilation of breast cancer without ER, PR and HER2 expression, which not only lacks effective therapeutic targets but also has great clinical and biological heterogeneity [35, 36]. Major efforts have been made to categorize TNBC heterogeneity and develop corresponding target therapies, which are significant to improve TNBC clinical outcomes. [4–6, 37, 38]. Among TNBC subtypes, CSC-enriched subtype remains a clinical conundrum with shorter recurrence-free survival compared to other subtypes. Our study revealed that DCLK1-medicated IL-6/STAT3 activation played an important role in promoting CSC traits, reducing sensitivity to chemotherapeutics, and favoring immune escape by reducing cytotoxic T cells infiltration, which provided potential targets for TNBC personalizing treatment.

A recent study classified TNBC into four types according to the genomic, transcriptomic and clinical data of a cohort of 465 TNBC patients, including luminal androgen receptor (LAR), immunomodulatory (IM), basal-like immune-suppressed (BLIS) and mesenchymal-like (MES) [6]. Compared to previous TNBC classifications, the newly identified TNBCtype-4 classification took full account of intratumoral immune components, with the improving understanding of the crosstalk between tumor cells and their microenvironment. The corresponding treatment strategies for each subtype were designed and experimented subsequently [37]. The MES subtype, which is enriched in stem-like cells and CSC-related signaling, still lacks suitable therapeutic options due to the absent of CSC-specific targets in TNBC. Here, our study disclosed that DCLK1 was not only significantly and highly expressed in MES subtype but also an important driver of TNBC stemness. As a newly identified gastrointestinal tumor stem cell marker, DCLK1-related functions in tumor malignant progression have been gradually revealed in the past decade [10, 12, 39]. In our study, we found that DCLK1 overexpression significantly promoted the CSC-like traits of TNBC cells including increased stem-like subpopulations and metastatic potential in MDA-MB-468 cells, while knockout of DCLK1 in BT549 cells showed the opposite effects. Innate resistance to chemotherapeutics is an important feature of tumor stem cells [40, 41]. Our study revealed that DCLK1-promoted stemness significantly decreased the sensitivity of TNBC cells to chemotherapy drugs including cisplatin and doxorubicin, and DCLK1 knockout increased the sensitivity, which suggested the combination of DCLK1 inhibitor with chemotherapeutics might be a promising therapeutic strategy for TNBC patients with drug resistance and tumor recurrence. In addition, with the renaissance of immunotherapy, the importance of tumor immune environment in anti-tumor therapy has been realized and the interactions between CSCs and the tumor immune environment have also been reviewed to better understand CSC-targeted therapies [42, 43]. Our previous study pointed out that DCLK1 affected antitumor immunity in pancreatic cancer [44]. Therefore, the impacts of DCLK1 on anti-tumor immunity in TNBC were investigated by 4T1 syngeneic mouse model. Our results revealed that DCLK1 knockout significantly inhibited subcutaneous tumors growth in immunocompetent mice but not immunocompromised nude mice, which implied the strong negative correlation between DCLK1 and anti-tumor immune. Tumor immune cell infiltration analysis by flow cytometry revealed that more cytotoxic T cells could be detected in DCLK1-knockout tumors and the deletion of CD8+ T cells could restore the growth of DCLK1-knockout tumors. TIDE analysis demonstrated that TNBC patients with higher DCLK1 expressions exhibited lower responsive to ICIs; these data suggest that inhibition of DCLK1 could both increase sensitivity to chemotherapy and activate antitumor immunity in TNBC.

The molecular mechanism of DCLK1 driving TNBC progression was further explored. GSVA analysis suggested a strong positive correlation between high DCLK1 expressions and IL-6/STAT3 signaling activation, which was verified in our subsequent cell experiments. Moreover, inhibiting this pathway in DCLK1-overexpressing cells using IL-6R antagonist, Tocilizumab or STAT3 inhibitor, S31-201 could block DCLK-induced malignant behaviors in TNBC. The aberrant activation of IL-6/JAK/STAT3 signaling occurs in many types of tumors and targeting this signaling for the treatment of solid tumor has been clinically investigated [45–47]. In breast cancer, higher expression of IL-6 and DCLK1 predicts shorter recurrence-free survival in TNBC but not in luminal A, luminal B and HER2 subtypes, which suggests the critical roles of DCLK1 and IL-6 in tumor recurrence of TNBC. Moreover, DCLK1 was significantly and highly expressed in the MES subtype of TNBC, and a study from Shen Zhao et al. firstly pointed out that DCLK1 could be a clinical representative marker for this subtype [20]. These data revealed the potential of DCLK1 in treating TNBC, especially for the MES subtype. Then, we found that DCLK1 specific inhibitor, DCLK1-IN-1, sensitized the mesenchymal-like cells BT549 to chemotherapeutics doxorubicin, while this effect was nonexistent in basal-like cells MDA-MB-468, and DCLK1-IN-1 also inhibited the stemness and metastatic potential of BT549 cells. Moreover, in vivo results revealed that inhibition of DCLK1 increased the infiltration of CD8+ T cells (Figs. 3, 6), which suggested that DCLK1 inhibitor was conducive to alter immunologically cold tumor to immunologically hot tumor benefiting from immunotherapy. These results revealed that inhibition of DCLK1 in the MES subtype could be a promising treatment by increasing cellular sensitivity to chemotherapeutics and activating the antitumor immunity.

In summary, targeting DCLK1-mediated IL-6/STAT3 pathway might be a promising treatment strategy for the TNBC patients with high DCLK1 expression, especially for the MES subtype, which is significant to targeted therapies based on TNBC subtypes.

## Supplementary Information

**Additional file 1: Fig. S1**. The proportion of CD8+ T cells was unchanged in thymus after anti-CD8α treatment..

**Additional file 2: Fig. S2**. The activation of IL-6/STAT3 pathway promotes the CSC-like phenotypes of TNBC cells.

## Data Availability

All data in this study were presented in the articles.
