# Supplementary figures and images for: Targeting DCLK1 attenuates tumor stemness and evokes antitumor immunity in triple-negative breast cancer by inhibiting IL-6/STAT3 signaling
Source: Breast Cancer Res. 2023 Apr 17;25:43. doi: 10.1186/s13058-023-01642-3 (PMC10108533; doi:10.1186/s13058-023-01642-3)

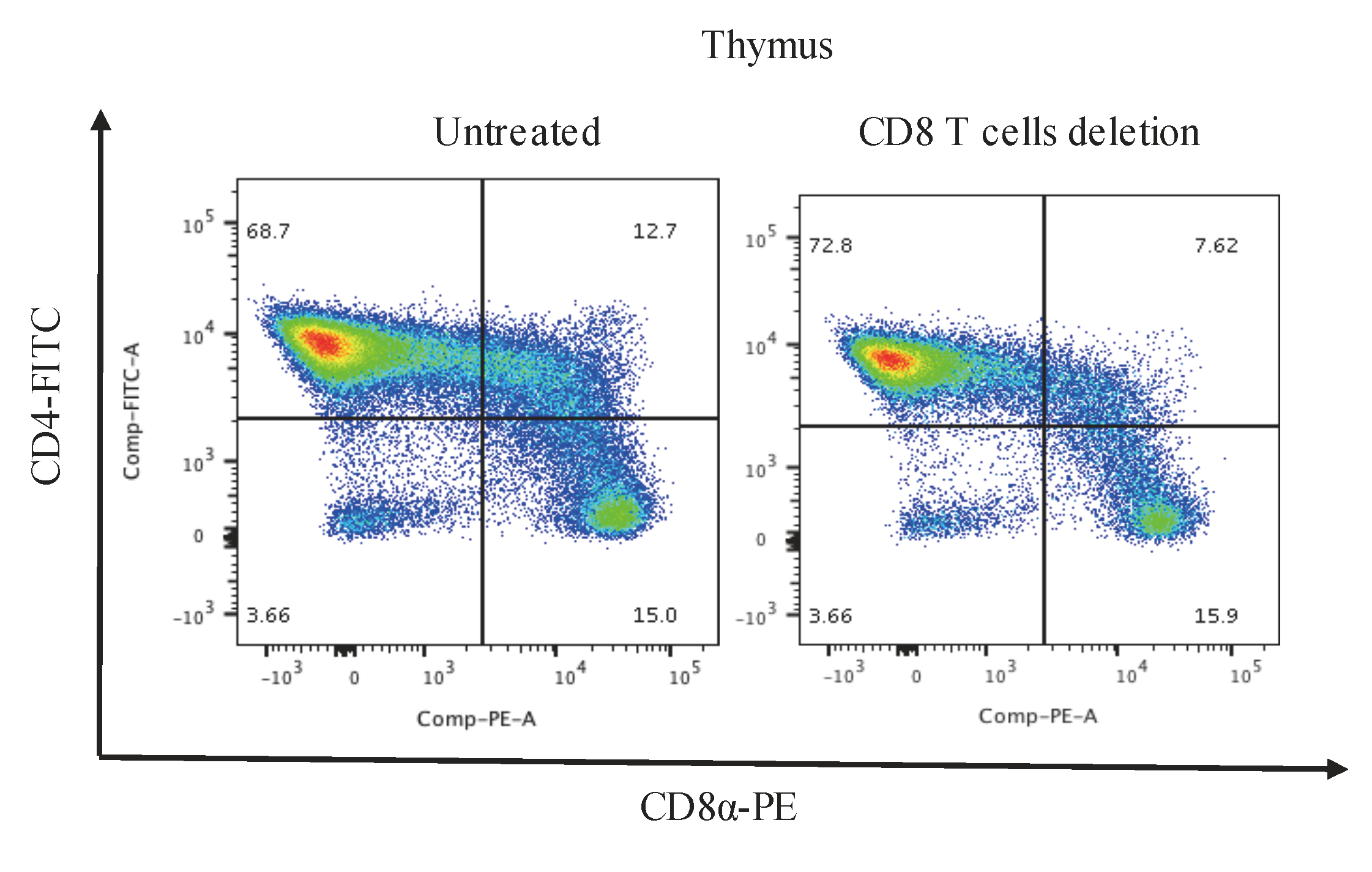

Supplement: Supplementary file 1 — Additional file 1: Fig. S1. The proportion of CD8+ T cells was unchanged in thymus after anti-CD8α treatment.. [file 13058_2023_1642_MOESM1_ESM.tiff]

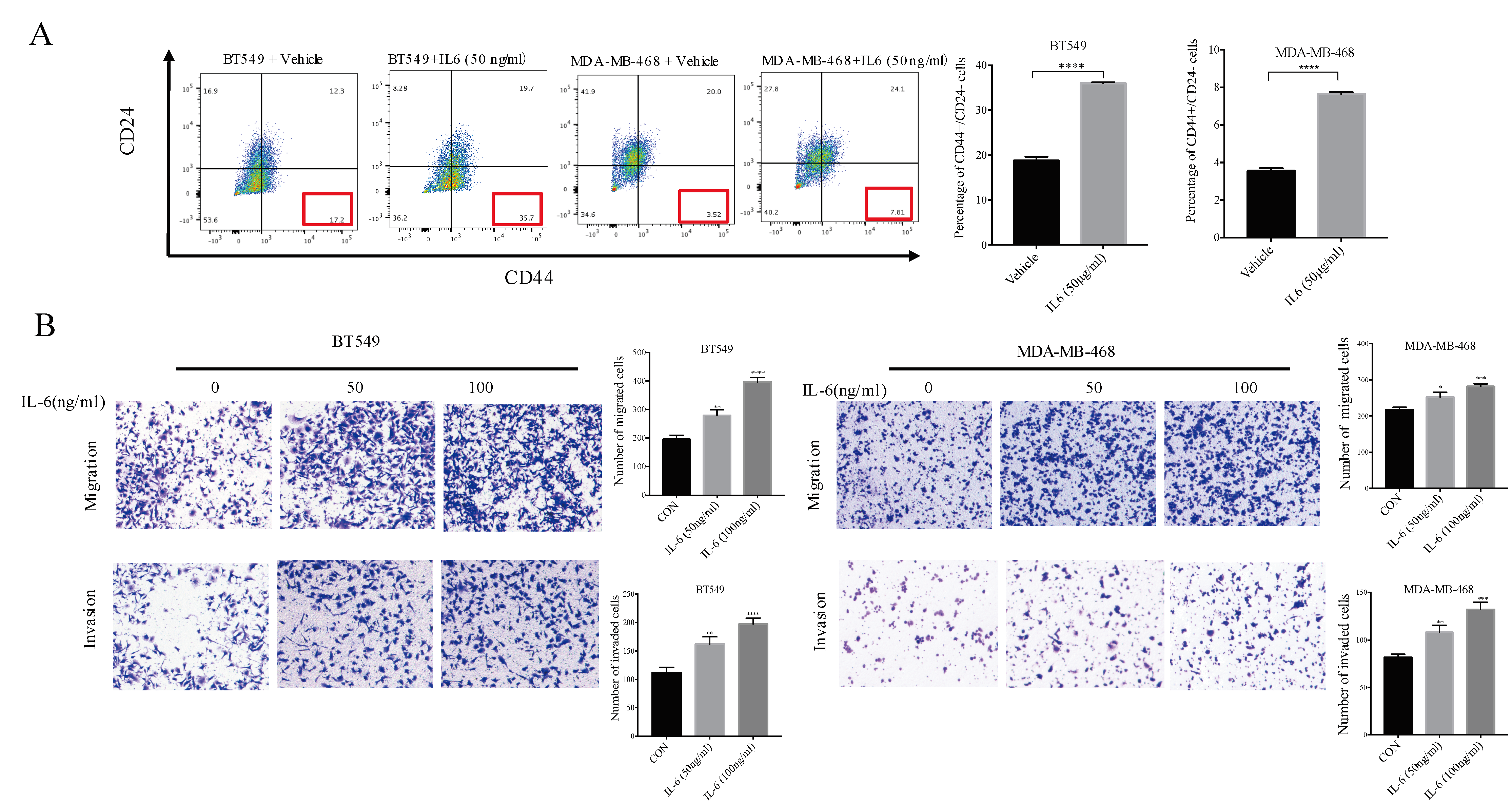

Supplement: Supplementary file 2 — Additional file 2: Fig. S2. The activation of IL-6/STAT3 pathway promotes the CSC-like phenotypes of TNBC cells. [file 13058_2023_1642_MOESM2_ESM.tiff]
